# Letter to the Editor: ‘Musculoskeletal manifestations of diabetes mellitus – an update’

**DOI:** 10.1016/j.clinme.2026.100569

**Published:** 2026-03-19

**Authors:** Vaishali Khobragade, Prajakta Udmale, Aparna Dixit

**Affiliations:** Department of Human Anatomy, Dr. DY Patil Homoeopathic Medical College and Research Centre, Dr. DY Patil Vidyapeeth (Deemed to be University), Pimpri, Pune, India

To the Editor

We read with great interest the recent review by Ward and Jawad titled *‘Musculoskeletal manifestations of diabetes mellitus – an update’*.

The article highlights the rise in musculoskeletal difficulties related to diabetes mellitus (DM), which is expected to affect 1.31 billion individuals by 2050. It focuses on lesser-known disorders such as diabetic cheiroarthropathy, amyotrophy, sarcopenia and widespread idiopathic skeletal hyperostosis, and it introduces new treatments such as glucagon-like peptide-1 receptor agonists and denosumab.^1^

The review methodically categorises joint, soft tissue, muscle and bone problems, which is useful for general practitioners and rheumatologists. It emphasises the link between musculoskeletal dysfunction and insufficient glycaemic management, citing epidemiological data that reveal a 58% incidence of musculoskeletal illnesses among patients with diabetes. Furthermore, it tackles fracture risk factors other than bone mineral density for type 2 diabetes, emphasising the importance of bone quality in measuring fragility risk.^2^, ^3^

While the summary of advanced glycation end-products and chronic inflammation in musculoskeletal degeneration is useful, it falls short in discussing the connections between insulin resistance, adipokine dysregulation and mitochondrial dysfunction. The study focuses on the function of metabolic syndrome-related inflammatory pathways in sarcopenia and osteoarthritis, regardless of obesity.^4^ Furthermore, while glucagon-like peptide-1 receptor agonists show promise, their therapeutic utility remains unknown, based mostly on preclinical trials rather than musculoskeletal outcomes.^5^ To improve therapeutic relevance, distinguish between hypothesis and evidence-based recommendations.

FRAXPlus and QFracture are two approaches to fracture risk stratification, although there are no particular guidelines on screening intervals or DXA^1^ criteria for people with type 2 diabetes, who have a higher fracture risk while having normal bone mineral density.^3^ The research also discusses the relationship between rheumatoid arthritis and stiff person syndrome, recommending genome-wide association data to better understand the immunogenetic links between type 1 diabetes and other autoimmune rheumatic disorders.

This study emphasises the importance of musculoskeletal difficulties in diabetes and calls for a multidisciplinary approach. It proposes improvements in mechanistic integration, unambiguous evidence stratification for new treatments, and realistic diabetic screening algorithms to reduce the expected worldwide diabetes^1^ burden while improving long-term metabolic and functional outcomes.

## CRediT authorship contribution statement

**Vaishali Khobragade:** Conceptualization, Methodology, Writing – original draft. **Prajakta Udmale:** Supervision, Validation, Writing – original draft. **Aparna Dixit:** Writing – review & editing.

## Funding

No funding was received for the preparation of this letter to the Editor. The authors did not receive any financial support from public, commercial or not-for-profit funding agencies for the research, authorship or publication of this work.

## Declaration of Competing Interest

The authors declare that they have no known competing financial interests or personal relationships that could have appeared to influence the work reported in this letter. The authors have no financial or non-financial conflicts of interest to disclose.

## Footnotes

^1^ DXA (Dual-Energy X-ray Absorptiometry) is a low-dose X-ray diagnostic test that measures bone mineral density to predict fracture risk, even in circumstances such as Type 2 Diabetes where bone density seems normal.
